# Low Temperature Bonding by Infiltrating Sn3.5Ag Solder into Porous Ag Sheet for High Temperature Die Attachment in Power Device Packaging

**DOI:** 10.1038/s41598-018-35708-6

**Published:** 2018-11-27

**Authors:** Chunjin Hang, Junjian He, Zhihao Zhang, Hongtao Chen, Mingyu Li

**Affiliations:** 1 State Key Laboratory of Advanced Welding and Joining, Harbin Institute of Technology, Harbin, 150001 China; 2 Department of Materials Science and Engineering, Harbin Institute of Technology at Shenzhen, Shenzhen, 518055 China; 3 Fujian Key Laboratory of Advanced Materials, Department of Materials Science and Engineering, College of Materials, Xiamen University, Xiamen, 361005 China

## Abstract

We have proposed a high temperature die attach method with porous Ag sheet as an interlayer for power device packaging. Sn-3.5Ag solder paste can infiltrate into the porous Ag sheet through capillary forces and Sn can react with the porous Ag sheet and Ag metallizations at the interfaces to form Ag_3_Sn after reflow at 260 °C for 10 min. The large specific surface area and the high diffusion rates between Ag and Sn accelerate the Sn consumption in the porous Ag structure, thus significantly reducing the processing time. The difference of the melting points of the die attach material before and after reflow could be expanded as large as 259 °C. The bondlines show good electrical and thermal conductivities. Furthermore, the average shear strength of the bondlines at 300 °C is higher than 20 MPa. The porous Ag skeleton remained in the bondline would contribute greatly to the heat dissipation and the electrical signal transmission in power devices.

## Introduction

SiC and GaN, known as the third-generation semiconductors, become the most promising replacements for Si semiconductor due to their excellent physical properties, such as wide band gap, high saturated electron drift velocity, and high critical breakdown field^1,2^. In particular, SiC semiconductors are able to operate properly at high temperatures up to 600 °C without sacrificing its physical properties. As a consequence, they can be used in harsh working conditions, e.g. high power, high temperature, high frequency, and high radiation environments^3–8^. This would be of great help to the industries of down-hole drilling, space exploration, automobile, nuclear power, and so on. Unfortunately, no appropriate die attach material can withstand the harsh environments and make full use of the excellent properties of the third-generation semiconductors till now.

The melting points of traditional high-lead solder pastes are approximately 300 °C, which are fit for die attachment in power device packaging. However, they are gradually being phased out due to the hazardous effects of Pb on human beings and the environment^9^. Though some Au-containing solder alloys, such as Au80Sn20, Au12Ge and Au3Si, can provide mechanical attachment at high temperatures, their high costs have limited their further development and applications^10^. Bi-Ag and Zn-Al solder alloys can also form high temperature interconnections. Unfortunately, the low thermal conductivity and the susceptibility to oxidation and corrosion are their insurmountable inherent limitations, respectively^11–13^. In addition, the high processing temperatures required by the high melting points of the die attach materials tend to introduce large residual stresses in bondlines generated by the coefficient of thermal expansion (CTE) mismatches between different packaging materials during solidification, leading to the deteriorations of the mechanical strength and the thermo-mechanical performance, or even premature failure. As a consequence, the die attach materials applicable for high power device packaging require a low processing temperature to reduce residual stress but a high remelting temperature for high temperature service.

Currently, two most promising approaches to solving this problem are nano-silver sintering^14–16^ and transient liquid phase (TLP) bonding^17–19^. Nano-silver paste sintering can reduce the processing temperature by using the large specific surface area and high surface energy of nano-silver particles to depress the melting point. After sintering, the nano-silver particles can interconnect with each other and recover to the melting point of bulk Ag (961 °C)^20^. In fact, voids are hard to be completely eliminated from the nano-silver sintered bondlines because of the volatilization of organic solvents, especially for those dies with large dimensions. The voids can degrade the electrical performance, thermal dissipation ability, and reliability of power devices^21,22^. In addition, the toxicity of nano-silver particles is still unknown and the high electromigration tendency of Ag may seriously affect the reliability of the formed bondlines. In recent years, TLP bonding technique has received increasing attention in academic and engineering fields because it can make a high remelting point bondline at a low processing temperature with a low cost^23–27^. TLP bonding method needs a low-melting-point metal sandwiched between two high-melting-point metals, and isothermal solidification after reflow makes the low-melting-point metal react with the high-melting-point metals to form intermetallic compounds (IMCs), which usually have much higher remelting temperatures than the low-melting-point metal. When the low-melting-point metal was depleted, the bondline can service at a much higher temperature than the processing temperature. So far, many TLP systems have been developed, such as Cu-Sn^28^, Ni-Sn^29^, Ag-Sn^27,30^, and Ag-In^31^. However, the growth fronts of IMCs are usually not flat, and a very long reflow time is required to make sure no low-melting-point phase left in the gaps after IMCs grown from both sides impinged. TLP bondlines are composed of thin IMCs and exhibit low reliability because of their poor ability to absorb stress. Recently, high-melting-point metal powders blended with low-melting-point metal powders have been developed as a TLP composite paste to accelerate the consumption of the low-melting-point phase^32–35^. However, it is hard to obtain a uniform microstructure in the resulting bondline because the low-melting-point metal powders tend to aggregate after melting.

In addition to the above mentioned drawbacks, both nano silver sintering and TLP bonding techniques require large pressure to assist bonding, making the process more complicated and inconvenient to be integrated into the existing production lines. As a consequence, an appropriate pressureless or low-pressure die attach process with a high remelting point is urgently needed for the packaging of the third-generation semiconductors. In this study, we propose a low-pressure bonding process at low temperature to make Sn3.5Ag solder infiltrate into the porous Ag, and a bondline with Ag-Sn IMCs entangled with Ag can be fabricated within a short reflow time. The porous Ag sheet not only provides the large specific surface area to accelerate the consumption of the low-melting-point phase, Sn, but also ensures a high thermal conductivity and a low electrical resistance, which are the most desirable characteristics of die attach materials. At the same time, the bondline can sustain a temperature as high as 480 °C when the Sn in Sn3.5Ag solder was consumed to form the high remelting Ag-Sn IMCs. Furthermore, the die attach method is compatible with the existing reflow facility and process because it can be performed at 260 °C under a small pressure of 0.5 MPa.

## Experimental

**Figure 1:**
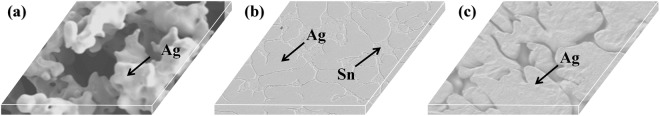
SEM images of porous Ag sheets (**a**) microstructure before electroplating (**b**) cross-section of compressed Sn-electroplated porous Ag (**c**) microstructure of compressed Sn-electroplated porous Ag after corrosion using dilute hydrochloric acid.

Pure Sn was electroplated on a commercial porous Ag sheet (10 mm × 10 mm × 500 μm) with pore sizes ranging from 80 μm to 150 μm. The electroplating solution consists of 70~90 g·L^−1^ sodium stannate, 8~15 g·L^−1^ sodium hydroxide, 15~25 g·L^−1^ sodium acetate, and 1.34~1.78 g·L^−1^ hydrogen peroxide. Porous Ag sheet was electroplated in the electroplating solution at 60~80 °C for 20 min. The Ag sheet after Sn electroplating was compressed into a sheet with a thickness around 150 μm, as shown in Fig. 1(a–c). The Sn in the compressed sheet was then removed completely using the dilute hydrochloric acid. By this step, the pores in the porous Ag sheet were compressed into slits with width about 300 nm, which is an appropriate size to deplete the infiltrated Sn in a short reflow time. If the commercial porous Ag sheet was compressed directly without electroplating Sn, the pore sizes after compression are not uniform with large distribution range, which is not fit for rapid Sn consumption. The Sn3.5Ag solder paste was stencil printed on two Cu substrates (10 mm × 10 mm × 3 mm) coated with an Ag layer with a thickness of 15 μm, and then the prepared porous Ag sheet was sandwiched between the two substrates as the interlayer. The sandwich samples were reflowed on a heating stage at the temperature of 260 °C for 3 min, 5 min, 8 min and 10 min, respectively. The bonding process was performed under air atmosphere with 0.5 MPa pressure.

For the sake of comparison, a Sn interlayer with a thickness of 20 μm was also sandwiched between two Ag coated Cu substrates and reflowed at the same temperature to make Ag-Sn TLP bondlines as a benchmark. Shear testing was performed by a creep testing machine (GWTA-105) on the bondlines fabricated with the porous Ag sheets and the traditional Ag-Sn TLP bonding technique. The shear rate was set to be 0.2 mm/min. To examine the microstructures of the porous Ag sheets (PAS) bondlines, the cross-section samples were prepared by grinding and polishing using the routine metallographic method. A scanning electron microscope (SEM, Hitachi-S4700) with an attached electron dispersive x-ray detector (EDX, EDAX XM4) was adopted to observe and analyze the microstructures. X-ray diffraction (XRD, Rigaku D/max 2500) was used to analyze the compositions of different phases in the bondlines. Five samples in each group were adopted for shear testing and the average was reported as the shear strength. The thermal conductivity (*k*) was calculated from *k* = *α ∙ ρ ∙ c*. The thermal diffusivity (*α*) was measured by laser flash diffusivity method (Netzsch LFA477); the samples adopted were prepared by reflowing the compressed porous Ag sheets with Sn3.5Ag solder, and the reflowed samples were cut into dimensions of 10 mm × 10 mm × 1.4 mm for thermal diffusivity measurement. We choose 30 °C and 300 °C as the testing temperatures, and at least five samples were tested at each temperature. The maximum and minimum values were removed and other results were provided; the density (*ρ*) were determined by Archimedes drainage method; the melting points and the specific heat capacities (*c*) were measured by differential scanning calorimetry (DSC, Netzsch STA 449F3). The electrical resistivities of the bondlines were determined by the four-probe method.

## Results and Discussion

### Microstructure characterization

**Figure 2:**
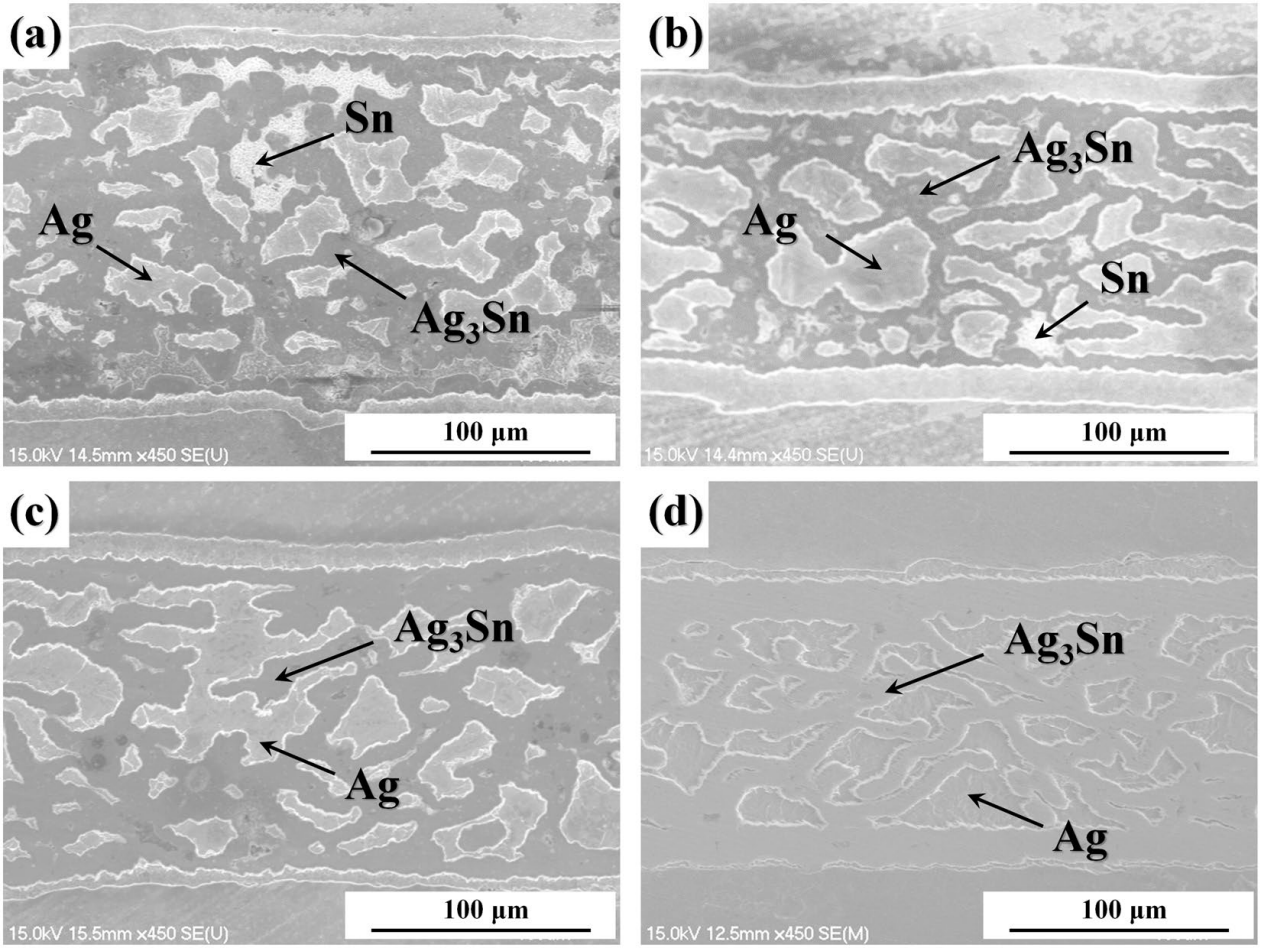
Cross-sections of bondlines fabricated by porous Ag after reflowed at 260 °C for (**a**) 3 min (**b**) 5 min (**c**) 8 min (**d**) 10 min without applying pressure.

The microstructure of the reflowed bondline was shown in Fig. 2. For better observation, the bondline was immersed in 20 mL 10 vol.% HCl solution for 3 s and then etched by a mixture of 10 mL 25~28 wt.% NH_3_ ·H_2_O and 10 mL 30 wt.% H_2_O_2_ solution for 10 s to reveal the microstructure of Sn and Ag, respectively. The Sn3.5Ag solder paste can melt, wet and spread on the surfaces of the porous Ag and the Ag metallizations at the interfaces when the reflow temperature was increased above its melting point (221 °C). The pores in the porous Ag can be filled up with the molten solder due to the capillary force. In this way, the reflow process can be performed under a low pressure of 0.5 MPa, increasing the possibility of industrialization with the ease of operation and compatibility with the existing process and facilities. Residual Sn still can be observed in the bondlines after reflow at 260 °C for 3 min and 5 min, as shown in Fig. 2(a,b), respectively. As the reflow time was extended to 8 min, as shown in Fig. 2(c), Sn almost disappeared and the volume fraction of Ag was decreased gradually as the reaction between Ag and Sn continued. After reflow at 260 °C for 10 min, as shown in Fig. 2(d), almost all Sn has been consumed to form Ag-Sn IMCs. The EDS results show that the reaction product formed at the interfaces with the porous Ag and the Ag substrates is Ag_3_Sn, which has a high remelting point of 480 °C. That is to say, the reflowed bondlines have an ability to sustain a much higher temperature than the processing temperature with the newly formed Ag-Sn IMCs. The difference of the melting points of the die attach material before and after reflow could be expanded as large as 259 °C. The low processing temperature will decrease the die level stresses after reflow and minimize the thermal damage to other devices or substrates in the system brought by the heating process. It has been reported that creep was one of the serious concerns for the reliability of lead-free solder interconnections during service because the homologous temperature of lead-free solder is greater than 0.5 even at room temperature^36^. The bondline fabricated in this work can increase the remelting temperature and thereby decrease the homologous temperature, improving the reliability of bondlines during service at elevated temperatures. Estimated from the infiltation condition of Sn3.5Ag solder in the porous Ag sheet from the cross-section, the void ratio should be below 5%. No large void was observed in the bondline, and it would contribute greatly to the thermal and electrical performances and reliability of the power devices.

**Figure 3:**
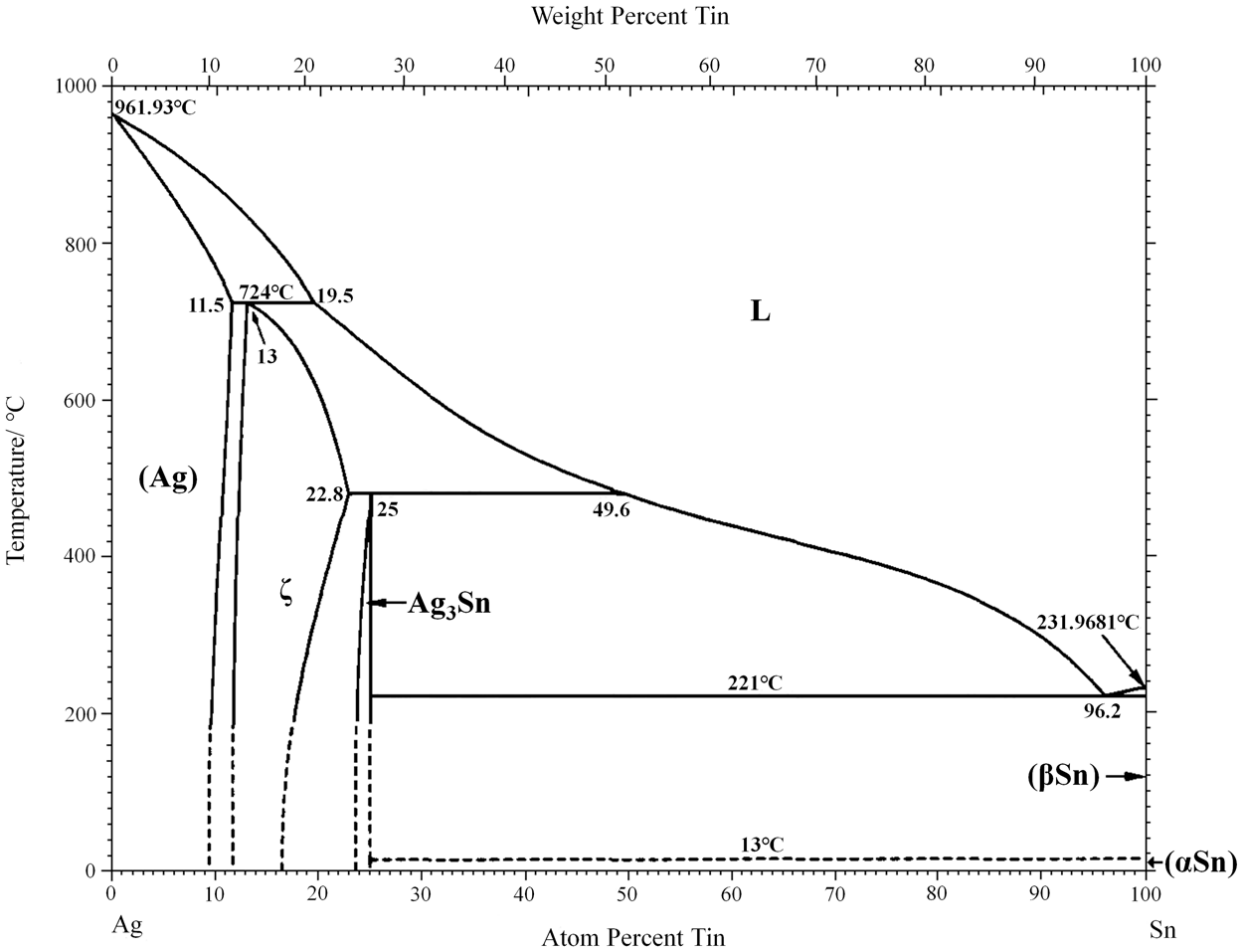
Ag-Sn binary phase diagram.

**Figure 4:**
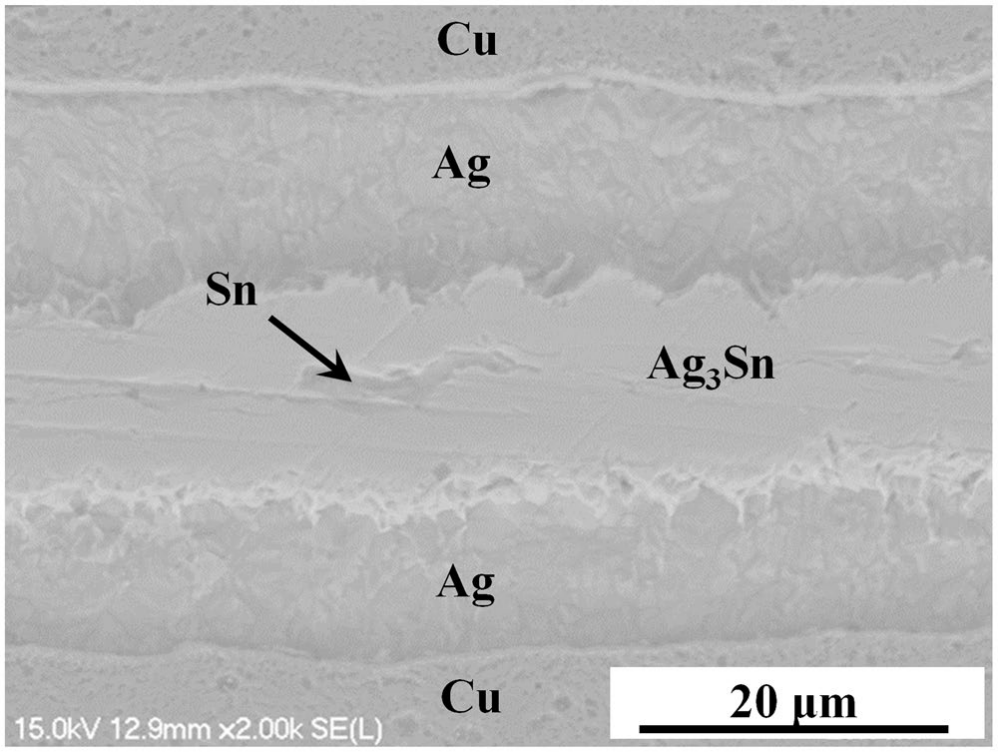
Cross-sections of TLP bondline after reflowed at 260 °C for 30 min.

More Ag was consumed to form Ag-Sn IMCs as the reflow time was increased. Theoretically, if the reflow time extends further, the formed bondline could withstand a much higher temperature up to 724 °C because the reaction products will turn into Ag (Sn) solid solution according to the Ag-Sn binary phase diagram, as shown in Fig. 3. In fact, the porous Ag sheet offers a much larger reaction area for Ag and Sn compared with the planar reactions in traditional TLP bonding. Furthermore, the dissolution and diffusion of Ag in Sn is nearly as high as that of Au^37^. As a consequence, the consumption rate of the low-melting-point Sn was significantly accelerated in the PAS bondlines. In contrast, the low-melting-point Sn phase with a thickness of only 20 μm cannot be consumed completely and still remained in the TLP bondline after reflowed at 260 °C for 30 min, as shown in Fig. 4. This phenomenon was in good agreement with the reported conclusion in other ref.^38^.

**Figure 5:**
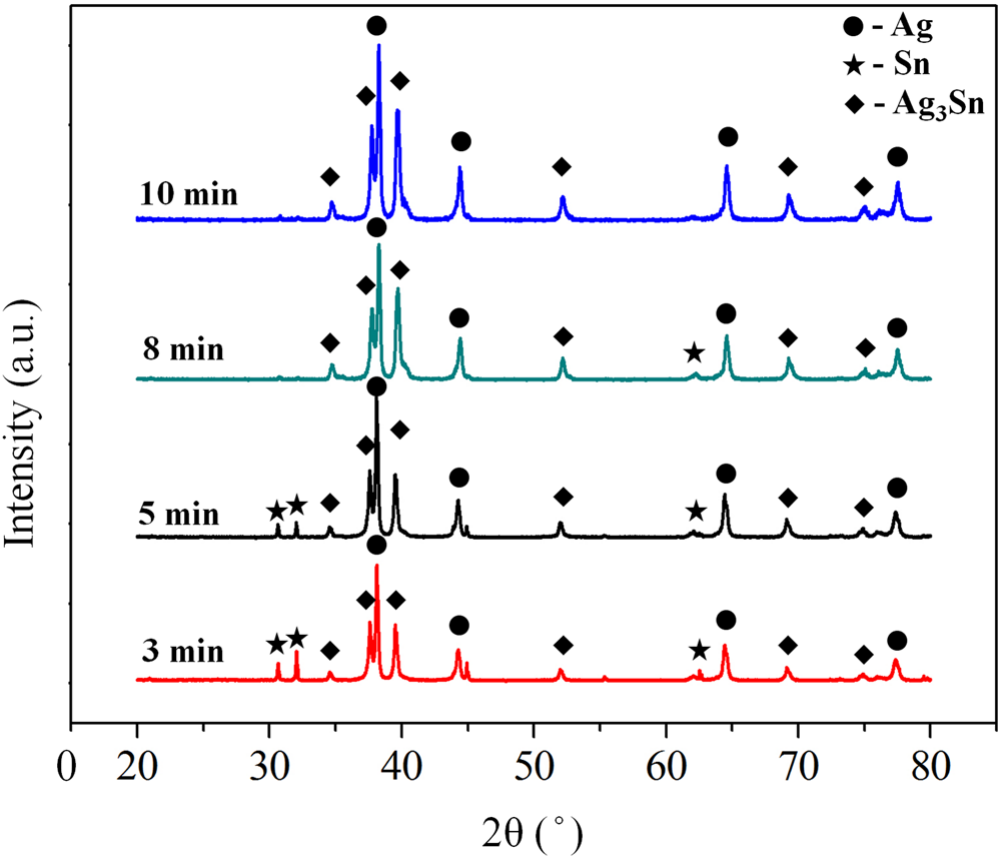
XRD patterns of compressed Sn-electroplated porous Ag samples after different reflow times.

As shown in Fig. 5, XRD was adopted to confirm the phase compositions of the Sn-plated porous Ag after reflow for different times. After reflow for 3 min, the XRD peaks correspond to Ag, Sn, and Ag_3_Sn. With the increasing reflow time, Sn was gradually consumed to form Ag_3_Sn. After reflow for 10 min, the Sn peaks are hard to be discerned. In other words, the highest temperature that the bondline can withstand has been increased to 480 °C, which is the melting point of Ag_3_Sn.

### Electrical and thermal properties

The average electrical resistivity of the PAS bondline was measured to be 4.38 ± 0.06 μΩ·cm, which falls in between the electrical resistivities of Ag (1.65 μΩ·cm) and Ag_3_Sn (6.08 μΩ·cm). The porous Ag sheet remained in the bondline after reflow offers low electrical resistivity, which is much lower than that of Sn (11.50 μΩ·cm) and Sn3.5Ag (12.30 μΩ·cm). If the size of Ag ligaments and the amount of remained Ag can be controlled precisely, the electrical resistivity of bondline can be further decreased, and this part will be our future work. Similarly, the porous Ag sheet plays an important role in increasing the thermal conductivity of the formed bondline. The thermal conductivity of the die attach material is a crucial property for the reliability and the lifespan of power devices since the operation requires continuous heat dissipation during service. The thermal conductivity of the bondline is determined by thermal diffusion coefficient, density, and specific heat capacity, as given by the following equation:

1 $$k=\alpha \,\cdot \rho \cdot c$$

where, *k*–thermal conductivity (W·m^−1^·k^−1^); α–thermal diffusion coefficient (mm^2^/s); *ρ*–density (g/cm^3^); *c*–specific heat capacity (J·mg^−1^·K^−1^).

The thermal diffusion coefficient and the specific heat capacity at 30 °C were 29.60 ± 1.91 mm^2^/s and 0.25 ± 0.01 J·mg^−1^·K^−1^, respectively. At 300 °C, they were measured to be 30.04 ± 1.10 mm^2^/s and 0.23 ± 0.03 J·mg^−1^·K^−1^, respectively. The density was determined to be 9.68 g/cm^3^. According to equation (1), the thermal conductivities were calculated to be 71.63 ± 5.38 W·m^−1^·k^−1^ and 66.88 ± 5.91 W·m^−1^·k^−1^ at the temperatures of 30 °C and 300 °C, respectively. The thermal conductivity of the reflowed preform is much higher than that of the traditional Pb5Sn (35.6 W/m·K) solder alloy, which could contribute greatly to the heat dissipation of power devices during service^39^.

### Mechanical properties

**Figure 6:**
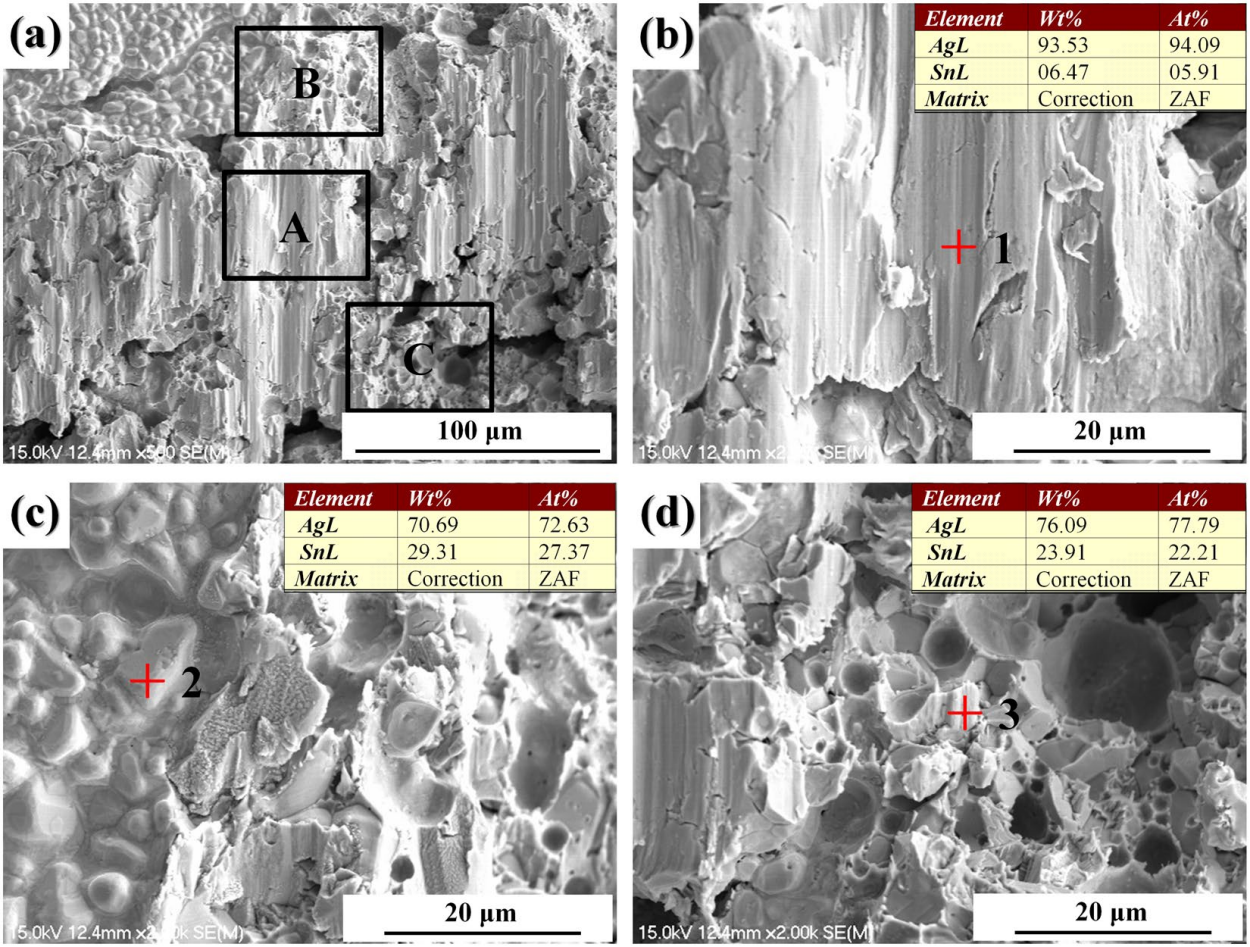
SEM images of fracture surfaces after shearing test: (**b**–**d**) are respectively high magnification images of area A, B, C in (**a**).

The average shear strength of the PAS bondlines at room temperature was measured to be 43.67 ± 4.89 MPa and the maximum shear strength could reach up to 47.62 MPa after reflow at 260 °C for 10 min, which satisfied the requirement of MIL-STD-883H method 2019.8. In contrast, it took at least 60 min to form an Ag-Sn TLP bondline by consuming the Sn interlayer with a thickness of 20 μm, and the average shear strength of the Ag-Sn TLP bondlines was measured to be 31.47 ± 3.11 MPa. To characterize the interconnection performance at high temperatures, the prepared PAS bondlines were subjected to shear testing at 300 °C and the average shear strength of the bondlines was determined to be 20.13 ± 5.28 MPa, which is sufficiently high for high temperature interconnection. As shown in Fig. 6(a), the typical fracture surfaces reveals the mixed mode of ductile and brittle fracture along Ag and Ag-Sn IMCs in the bondlines. Figure 6(b) shows plastic deformation in the remained Ag with obvious shearing trace. Scallop-like morphology of Ag_3_Sn and corresponding pits after shear testing were revealed, as shown in Fig. 6(c,d), respectively. Compared with other IMCs, Ag_3_Sn shows excellent ability to absorb stress with a low Young’s modulus of 78.9 GPa and a low hardness of 3.25 GPa; in fact, Ag_3_Sn is the most ductile IMCs among the common reaction products in electronic packaging, such as AuSn_4_, Cu_6_Sn_5_, Cu_3_Sn, and Ni_3_Sn_4_^40,41^. In addition, Ag_3_Sn shows the lowest electrical resistivity among them, which would contribute greatly to the overall electrical performance of the formed bondline^41^. That is to say, the porous Ag sheet infiltrated with Sn-Ag solder can be a promising high temperature die attach material with good thermal and electrical conductivities and mechanical properties.

## Conclusions

We report a die attach method by infiltrating Sn3.5Ag solder into the porous Ag sheet for high temperature power device packaging. The low-melting-point Sn3.5Ag solder decreased the processing temperature, and facilitated the bonding by wetting and spreading on the surfaces of the porous Ag through the capillary forces. The consumption of low-melting-point Sn was accelerated significantly by providing a large specific surface area for reactions by using the porous Ag sheet. The low-melting-point phases have been consumed to form the high remelting point Ag-Sn IMCs after reflow at 260 °C for 10 min, and then the bondline can withstand the elevated temperatures during high power device service. The average shear strength of bondlines at 300 °C is 20.13 ± 5.28 MPa. The thermal conductivities of PAS bondlines are 71.63 ± 5.38 W·m^−1^·k^−1^ and 66.88 ± 5.91 W·m^−1^·k^−1^ at the temperatures of 30 °C and 300 °C, respectively. The average resistivity of the bondlines is 4.38 ± 0.06 μΩ·cm. The Ag networks that remained in the bondlines after reflow increased the thermal conductivity and decreased the electrical resistivity, which would contribute greatly to the heat dissipation and electrical signal transmission in power devices. PAS bondlines with good thermal, electrical and mechanical properties provide a potential solution for die attachment in high power devices.
